# Effects of 4-Aminopyridine on Cloned hERG Channels Expressed in Mammalian Cells

**DOI:** 10.1111/j.1753-5174.2009.00021.x

**Published:** 2009-09

**Authors:** Muthukrishnan Renganathan, Serguei Sidach, Andrew R Blight

**Affiliations:** * Millipore Corporation, St. CharlesMO, USA; † Targacept, Inc, Winston-SalemNC, USA; ‡ Acorda TherapeuticsHawthorne, New York, USA

**Keywords:** 4-Aminopyridine (Fampridine), Cardiac Toxicity, Human *ether-à-go-go*-Related Gene (hERG), Human Embryonic Kidney 293 (HEK293) Cells, IC_50_ (Concentration Resulting in 50% Inhibition), I_Kr_ (Delayed Rectifier Potassium Current), Multiple Sclerosis, Potassium Channel Blocker, QT Interval Prolongation, Torsade de Pointes (TdP)

## Abstract

**Introduction:**

Non-clinical evaluation of a medication's potential to induce cardiac toxicity is recommended by regulatory agencies. 4-Aminopyridine (fampridine) is a potassium channel blocker with the demonstrated ability to improve walking ability in patients with multiple sclerosis. We evaluated the *in vitro* effects of 4-aminopyridine on the human *ether-à-go-go*-related gene (hERG) channel current, since hERG current inhibition is associated with QT interval prolongation—a precursor to torsade de pointes (TdP).

**Methods:**

4-Aminopyridine was evaluated in concentrations ranging from 0.1 mM to 30 mM in human embryonic kidney 293 cells stably transfected with the hERG gene; terfenadine 60 nM was used as a positive control.

**Results and Discussion:**

We observed concentration-dependent inhibition of hERG current with 4-aminopyridine doses between 0.3 and 30 mM. The concentration of 3.8 mM resulting in 50% inhibition (IC_50_) is approximately three orders of magnitude higher than expected therapeutic plasma concentrations, suggesting 4-aminopyridine has low potential for prolonging QT interval or inducing TdP.

## Introduction

4-Aminopyridine (fampridine) is a potassium channel blocker that has been evaluated for the improvement of walking ability in patients with multiple sclerosis (MS). Several studies have demonstrated that 4-aminopyridine has the ability to significantly improve lower extremity strength and walking speed, relative to placebo, in a proportion of patients with MS [1–4]. At millimolar concentrations, 4-aminopyridine is a broad-spectrum blocker of potassium channels, as has been demonstrated in laboratory studies [5,6]. Typical plasma concentrations that are obtained during clinical studies, however, fall into a lower range of less than 1 µM (94 ng/mL). The characteristics of potassium channels that are blocked at these low concentrations have not yet been determined. The neurological effects of the compound, however, are consistent with potassium channel blockade.

Although an assessment of the general safety of a therapeutic agent is incorporated into clinical trials, the potential for cardiac toxicity is a distinct concern that requires independent evaluation. This concern arises primarily from the ability of some non–anti-arrhythmic drugs to delay cardiac repolarization—an undesirable characteristic that can induce the development of such potentially fatal cardiac arrhythmias as torsade de pointes (TdP) [7,8].

Since TdP is almost always preceded by prolongation of the cardiac QT interval, QT interval prolongation is recognized as a surrogate marker for pro-arrhythmic risk [7,9]. Prolongation of the QT interval has been associated with the induction of TdP across a broad range of non-cardiac drugs [7,10]. Consequently, clinical assessment of the potential for QT interval prolongation is now part of the drug evaluation process required by the US Food and Drug Administration (FDA) for new drugs and for approved drugs when new indications, dosages, or routes of administration are introduced [9]. However, since prolongation of the QT interval is considered a less-than-optimal marker of cardiac risk [7,9], it has been additionally recommended that the potential cardiac toxicity of a drug be evaluated using non-clinical systems [11].

The rapid delayed rectifier potassium current (I_Kr_), of which the major channel protein is encoded by the human *ether-à-go-go*-related gene (hERG; K_V_11.1), is responsible for repolarization of the cardiac myocyte ventricular action potential [8,12,13]. Although moderate blockade of I_Kr_ results in the beneficial anti-arrhythmic effects of some cardiac drugs, inhibition of I_Kr_ is the most common cause of cardiac action potential prolongation by non-cardiac drugs leading to the development of TdP [10,14,15]. Whereas most, but not all, of the drugs associated with induction of TdP have also been shown to inhibit hERG channels [10,16], those that do inhibit these channels generally do so at concentrations that approximate therapeutic plasma levels [10].

The ability of human embryonic kidney 293 (HEK293) cells stably transfected with the hERG gene to produce a current analogous to I_Kr_[17] provides a reliable model for the *in vitro* evaluation of drugs for inhibition of I_Kr_. This model has been accepted as part of the non-clinical evaluation of a drug's potential for pro-arrhythmic effects [11,18].

Although a generally favorable safety profile has been reported for 4-aminopyridine in clinical trials [1–4], since the agent is a potassium channel blocker, it is important to determine its potential for cardiac toxicity through inhibition of the hERG potassium channel. The purpose of this study was to evaluate the *in vitro* effects of 4-aminopyridine on the hERG channel currents in a stably transfected human cell line.

## Methods

This study was conducted in accordance with Good Laboratory Practice Standards. All chemicals were obtained from Sigma-Aldrich (St. Louis, Missouri, USA.) unless noted otherwise.

### Cell Cultures

The cardiac potassium channel hERG gene was transfected into HEK293 cells (American Type Culture Collection, Manassas, Virginia, USA), an HEK cell line. This cell line was maintained at a low passage number (<50) at 37°C under 95% O_2_/5% CO_2_ atmosphere in Dulbecco's Modified Eagle's Medium/Nutrient Mixture F-12 (Invitrogen Corporation, Carlsbad, California, USA) supplemented with 10% fetal bovine serum (Invitrogen Corporation, Carlsbad, California, USA), 100 U/mL penicillin G sodium (Invitrogen Corporation, Carlsbad, California, USA), and 100 µg/mL streptomycin sulfate (Invitrogen Corporation, Carlsbad, California, USA); cell stocks were maintained in cryogenic storage. Transfection was performed using an adenovirus 5 plasmid–containing human hERG cDNA and the G418-resistance gene; 100 µg/mL G418 was added to the incubation medium after transfection to facilitate selection of stable colonies. Cells used for electrophysiology were plated in plastic culture dishes.

### Chemicals and Reagents

Test compounds included 4-aminopyridine (99.6% purity; obtained from Elan Pharma Ltd., Athlone, Ireland), terfenadine as a positive control, and the reference compound E-4031 (N-[4-[[1-[2-(6-methyl-2-pyridinyl)ethyl]-4-piperidinyl]carbonyl]phenyl] methanesulfonamide dihydrochloride), a high-affinity selective hERG blocker [8] that was used to subtract non-hERG current from each patch-clamp recording. Stock solutions of 4-aminopyridine were prepared in distilled water; stock solutions of terfenadine and the reference compound were prepared in dimethyl sulfoxide (DMSO) and stored frozen until use. Test formulations were prepared by dilution of stock solutions into a HEPES-buffered physiological saline (HB-PS) composed of 137 mM NaCl, 4 mM KCl, 1.8 mM CaCL_2_, 1 mM MgCl_2_, 10 mM HEPES, and 10 mM glucose, pH-adjusted to 7.4 with NaOH. Since DMSO at concentrations up to 0.3% does not affect hERG channel currents (Data on file, ChanTest Corporation), test solutions of the positive control and the reference substance contained 0.3% DMSO. Pipette solution for whole-cell recordings was composed of 130 mM potassium aspartate, 5 mM MgCl_2_, 5 mM EGTA (ethylene glycol tetraacetic acid), 4 mM ATP (adenosine triphosphate), and 10 mM HEPES, pH-adjusted to 7.2 with KOH. The pipette solution was prepared in batches, stored frozen at −80°C, and a fresh aliquot thawed each day.

### Electrophysiological Measurements

All experiments were performed at near-physiological temperature (35 ± 2°C), which was maintained using a combination of in-line solution heater, chamber floor heater, and feedback temperature controller, with the temperature measured using a thermistor probe immersed directly into a recording chamber. Patch pipettes were made from borosilicate glass capillary tubing using a P-97 micropipette puller (Sutter Instruments, Novato, California, USA). The pipette resistance was within the range of 2–5 MΩ. A commercial patch clamp amplifier was used for whole-cell recordings. Before digitization, records were low-pass filtered at one-fifth of the sampling frequency. Data acquisition and analysis were performed using pCLAMP software (version 8.2; Axon Instruments, Union City, California, USA).

Evaluation of 4-aminopyridine was conducted in two phases. The first phase was designed to identify the approximate concentration range and the second phase to determine the precise concentration-response relationship. In both phases, the onset and steady-state block of hERG channel currents were measured using a voltage protocol consisting of a 1-sec conditioning step to +20 mV, immediately followed by a repolarization test ramp (from +20 mV to −80 mV at 0.5 mV/second) applied at 5-second intervals from a holding potential of −80 mV.

For each experiment, cells were transferred to the recording chamber and superfused with HB-PS. Peak test pulse current was measured during the test ramp, and steady state was maintained for at least 20 second prior to application of 4-aminopyridine or the positive control. Peak current was measured until a new steady state was achieved. Each recording ended with a final application of a supramaximal concentration (500 nM) of the reference compound to assess the contribution of endogenous currents. The remaining unblocked current was digitally subtracted from all current traces to determine the potency of 4-aminopyridine for hERG current inhibition. The range of concentrations tested was 0.1–30 mM. Vehicle control and positive control experiments each were performed in three cells. Steady state was defined by the limiting constant rate of change with time, and steady state before and after each 4-aminopyridine application was used to calculate the proportion (%) of current inhibited at each concentration.

### Data Analysis

Concentration-response data were fitted to the following equation:


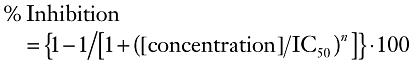


% Inhibition represents the proportion of hERG current inhibited at each concentration, IC_50_ is the concentration resulting in 50% inhibition, and *n* is the Hill coefficient. Non-linear least squares fits were performed with the Solver add-in for Excel 2000 (Microsoft Corporation, Seattle, Washington, USA) and the IC_50_ was calculated.

## Results

**Figure 1:**
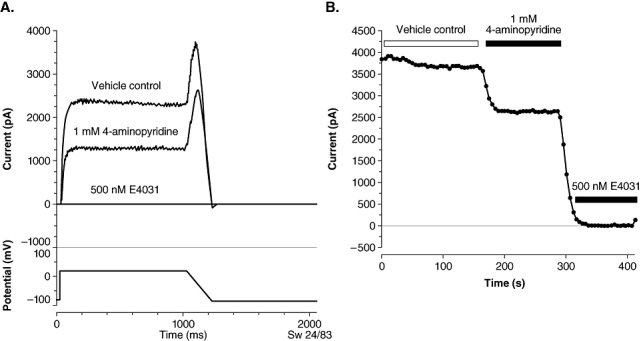
Sample traces of hERG current in the absence and presence of 4-aminopyridine (a), and during the time course of 4-aminopyridine evaluation (b). Measurements were performed at 35 ± 2°C using a voltage protocol consisting of a 1-sec conditioning step to +20 mV, followed by a repolarization test ramp (from +20 mV to −80 mV at 0.5 mV/s) applied at 5-s intervals from a holding potential of −80 mV.

Typical hERG voltage-clamp current traces acquired in control, after equilibration with 4-aminopyridine (1 mM), and after application of the reference compound E-4031 are illustrated in Figure 1a. Following activation by the conditioning prepulse, repolarization to −80 mV evoked a large, slowly decaying outward current, which was reduced with the application of 4-aminopyridine. The corresponding time course of the peak hERG current in control, and after applications of 4-aminopyridine and the reference substance, is shown in Figure 1b.

**Figure 2:**
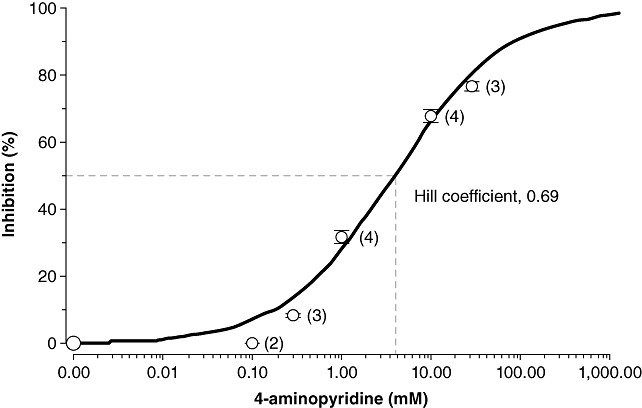
Concentration-dependent inhibition of hERG current by 4-aminopyridine. Numbers in parentheses indicate the number of replicates.

As demonstrated in the experimental data, 4-aminopyridine induced a concentration-dependent inhibition of the hERG channel currents. The mean percentage of current inhibited at each 4-aminopyridine concentration is presented in Table 1. The means (±standard deviation) for 4-aminopyridine concentrations of 0.3 mM, 1 mM, 10 mM, and 30 mM were 8.2 ± 0.9% (N = 3), 31.8 ± 3.8% (N = 4), 67.6 ± 3.6% (N = 4), and 76.7 ± 3.1% (N = 3), respectively. The concentration-response relationship for this effect is depicted in Figure 2. At the two lowest concentrations, 0.1 mM had no effect and inhibition of hERG by 4-aminopyridine 0.3 mM was minimal (8.2 ± 0.9%; N = 3). Increasing concentrations of 4-aminopyridine resulted in greater inhibition, with an estimated IC_50_ of 3.83 mM and a Hill coefficient of 0.69. For 0.3 and 1 mM 4-aminopyridine, steady state was reached within 1–1.5 minutes. For 10 and 30 mM, steady state was reached within 30 seconds. No steady state inhibition was reported for 0.1 mM since it had no inhibitory effect. Terfenadine, at a concentration of 60 nM, inhibited 82.8 ± 2.4% of the current (mean ± standard error; N = 3), confirming sensitivity of the test system to hERG inhibition.

**Table 1:** 4-Aminopyridine inhibition of hERG, expressed in stably transfected human embryonic kidney cells

4-Aminopyridine concentration, mM (number of replicates)	Mean percent inhibition (standard deviation)
0 (3)	0.3 (0.1)
0.1 (2)	0.3 (0.3)
0.3 (3)	8.2 (0.9)
1 (4)	31.8 (3.8)
10 (4)	67.6 (3.6)
30 (3)	76.7 (3.1)

## Discussion

This study used *in vitro* expression of hERG in a human cell line as a model for evaluating the effects of 4-aminopyridine on I_Kr_. This system has become a standard for non-clinical evaluation of the potential for QT interval prolongation [11,18], and the validity of the assay system in the current study was demonstrated by the positive control, terfenadine, which at a concentration of 60 nM resulted in approximately 82% inhibition. This high rate of inhibition is consistent with the hERG inhibition profile of terfenadine [16].

The data obtained with this system show that whereas 4-aminopyridine 0.1 mM has a negligible effect on hERG current, concentration-dependent inhibition occurs within the range of 0.3–30 mM. The calculated values for 4-aminopyridine with an IC_50_ of 3.83 mM and a Hill coefficient of 0.69 are comparable to those of 4.4 mM and 0.7, respectively, that have been reported by Ridley and colleagues using a similar assay system [19].

Although inhibition of hERG by 4-aminopyridine was observed, the clinical relevance of these data needs to be considered. A review of 100 pharmacological agents associated with induction of TdP reported that in general, drugs that present a risk for induction of TdP also inhibit hERG at concentrations close to those achieved in plasma during therapeutic administration [10]. A safety margin of at least 30-fold between the IC_50_ for hERG inhibition and the maximum plasma concentration achieved in clinical practice has been suggested as being adequate for predicting the safety of a drug with respect to risk for ventricular arrhythmias [10].

For 4-aminopyridine, the IC_50_ of 3.83 mM is several orders of magnitude higher than the maximum plasma concentrations that have been reported even at supratherapeutic doses. In a dose-ranging study of fampridine sustained-release (fampridine-SR) in patients with MS, which included a pharmacokinetic analysis, the therapeutic dose of 10 mg twice daily resulted in mean steady-state plasma concentrations of 0.243 ± 0.113 µM [2], which is 4 orders of magnitude lower than the IC_50_ for hERG. Even at the highest dose evaluated at 40 mg twice daily, the mean steady-state plasma concentration was 0.97 µM. Similarly, a study designed to evaluate the steady-state pharmacokinetics of fampridine-SR in patients with chronic spinal cord injury reported a mean maximum plasma concentration of 0.639 ± 0.160 µM with a 20-mg dose administered twice daily and 0.342 ± 0.095 µM with a 10-mg dose administered twice daily [20].

This greater than 4-log difference between the IC_50_ and therapeutic plasma concentrations suggests that the use of 4-aminopyridine is unlikely to result in QT prolongation and induction of TdP. However, the relationship of drug-mediated inhibition of hERG and the risk of TdP may be problematic. Nevertheless, the clinical risk for drug-induced arrhythmia and sudden death may be low, based on an analysis of adverse reactions of drugs with known anti-hERG activity, which showed that the higher the log of the ratio between therapeutic concentrations and the IC_50_, the lower the risk for cardiac adverse events [21]. However, an important consideration is that there may be individuals who achieve higher than expected peak plasma concentrations due to impaired metabolism, elimination, or overdosage. For perspective, a case report of substantial 4-aminopyridine overdose due to ingestion of 2–4 g resulted in plasma concentrations of 3.5 uM [22]. While such a high plasma concentration results in neurologic toxicity including seizure, it is still 3 orders of magnitude lower than the IC_50_ for hERG reported here.

These data suggest an electrophysiological basis for previous observations of a low risk of clinically significant cardiac effects associated with 4-aminopyridine. Clinical studies that have incorporated electrocardiogram (ECG) evaluation as a safety parameter reported no changes or trends in ECG values [2,20,23]. Additionally, in a study that evaluated the effects of a 1-month treatment regimen with 4-aminopyridine on cardiac parameters in patients with spinal cord injury, the cardiac parameters measured remained within normal range compared with literature-derived control values, and no evidence of QT prolongation was observed [24].

Although 4-aminopyridine exhibits dose-dependent inhibition of the hERG channel current, the calculated IC_50_ is approximately four orders of magnitude higher than reported therapeutic plasma concentrations. This difference suggests a margin of safety that may confer low torsadogenic potential based on the degree of I_Kr_ inhibition we observed. However, while I_Kr_ inhibition does play a prominent role in drug-induced TdP, which is the basis for the acceptance of hERG inhibition as an indicator of potential cardiac toxicity, it is important to recognize that the effects of drugs on other ion channels, such as increasing the plateau generated by sodium or calcium current, or decreasing other potassium currents, may also contribute to drug-induced QT prolongation.
